# Trap versus trade-off: cohort evidence on overeducation, income returns, and group disparities after China’s higher education expansion

**DOI:** 10.3389/fsoc.2026.1705158

**Published:** 2026-02-18

**Authors:** Yann Zhang, Yangyang Liu, Qing Yun, Junxiu Wang

**Affiliations:** 1 Institute of Sociology, Chinese Academy of Social Sciences, Beijing, China; 2 School of Marxism, Shandong University of Aeronautics, Binzhou, China; 3 Institute of Regional Education, China National Academy of Educational Sciences, Beijing, China; 4 School of Mental Health, Wenzhou Medical University, Wenzhou, China

**Keywords:** educational mismatch, higher education expansion, income return, overeducation, social inequality

## Abstract

**Background:**

Higher education expansion is a pivotal strategy for fostering economic growth and national competitiveness. Yet, its consequences for social equality—particularly through the mechanism of overeducation, where educational attainment exceeds job requirements—remain contested.

**Objective:**

This study investigates how the 1999 mass higher education expansion in China reshaped the incidence, income returns, and group disparities of overeducation by employing a theoretical framework of ‘trap versus trade-off.’

**Methods:**

Data were sourced from the Chinese General Social Survey (2010–2021). The study compared 19 birth cohorts that entered the labor market before and after the expansion. A total of 50,932 participants were included, aged 17–60 years and born between 1950 and 2003. Among all participants, 26,566 (52.16%) were female, and 19,166 (37.63%) were from rural areas. A cohort-based design by using the Hierarchical Age-Period-Cohort model was applied to resolve collinearity among age, period, and cohort effects.

**Results:**

The findings revealed four key insights. First, the expansion produced persistent cohort-level effects, embedding overeducation as a structural feature of the labor market. Second, the effect of expansion on overeducation interacted with other historical and policy shifts, highlighting the contextual nature of expansion outcomes. Third, and central to the study, we observed a transformation in the income effect of overeducation: it shifted from a trade-off (associated with income returns) for pre-expansion cohorts to a trap (associated with an income penalty) for post-expansion cohorts. Finally, the impact of overeducation on income returns varies across groups; while the aggregate trend reflects a trap, evidence suggests that overeducation may still function as a trade-off for certain disadvantaged groups, such as women and rural residents, potentially mitigating income inequality.

**Conclusion:**

These findings imply that to fulfill the fundamental aims of higher education expansion—raising educational attainment and reducing social inequality—policymakers must design targeted, group-sensitive interventions to address the unequal risks and opportunities generated during the expansion process.

## 1 Introduction

In 1999, the Chinese government decided to expand the higher education sector to meet demand for graduates stemming from a rapidly growing economy, cater to parents who want more higher education opportunities for their children, alleviate the problem of urban unemployment, and promote the development and utilization of China’s human resources (Wu and Zheng, 2008). Beginning in 1999, China made a significant leap forward in higher education (Knight et al., 2017).

**Figure 1:**
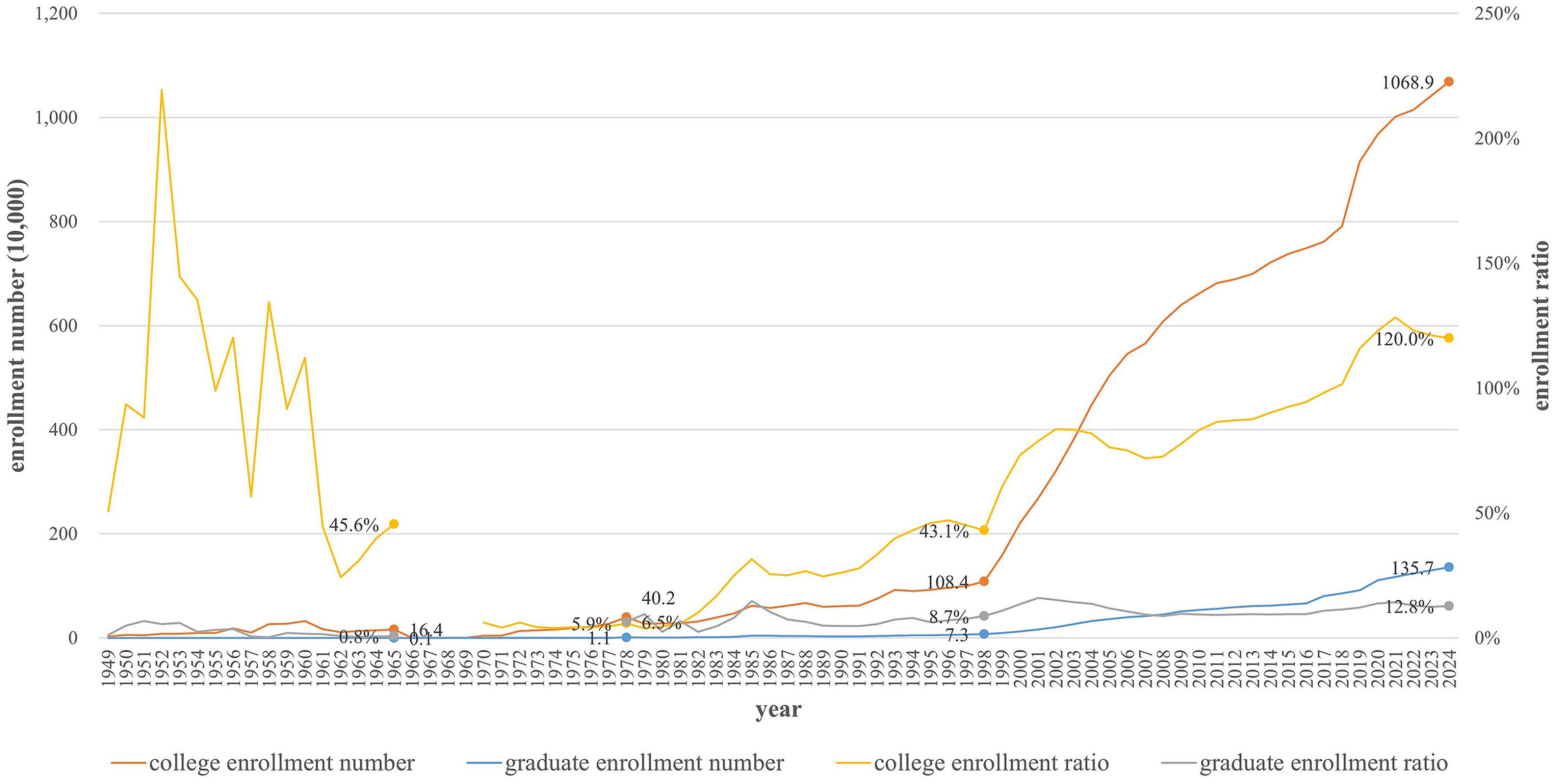
College and graduate enrollment number and ratio from 1949 to 2024.

Figure 1 shows the changes in enrollment numbers and rates from 1949 to 2024. Despite high fluctuations in the college enrollment ratio between 1949 and 1965, the college enrollment number, as well as the graduate enrollment number and ratio, were low. The restoration of college entrance exams (Gaokao) and the enrollment number and ratio of college and graduate education increased steadily from 1978 to 1998. The college enrollment was almost 2.5-folded, while the graduate enrollment was almost 7-folded. Nevertheless, because of the initially small enrollment number in 1978, that in 1998 remained relatively low. After the higher education expansion in 1999, there was rapid growth in college and graduate enrollment numbers and ratios. From 1998 to 1999, college enrollment increased almost 10-folded, from 1.084 million to 10.689 million; moreover, graduate enrollment increased almost 19-folded, from 73 thousand to 1.357 million. Additionally, the college enrollment ratio increased from 43.1 to 120%, and the graduate enrollment ratio increased from 8.7 to 12.8%.

The expansion of higher education in China appears to have achieved its mission of increasing higher education opportunities and promoting the development and utilization of human resources. However, an increase in underemployment or overeducation emerged (Wu and Zheng, 2008).

Overeducation is defined as educational attainment that exceeds the requirement of the job, represents a critical juncture where human capital investment may not translate into expected labor market returns (Zheng et al., 2021). Capsada-Munsech (2017) summarized overeducation incidence across countries from different periods; the percentage of overeducation ranged from over 10% to approximately 50%, with China at around 20%, showing a relatively high rate of overeducation globally. While its incidence has been widely documented, the fundamental nature of overeducation—whether it functions as a strategic trade-off for long-term career advancement or a persistent trap leading to enduring penalties—remains a pivotal theoretical and empirical debate.

Building upon the “trap versus trade-off” dichotomy, existing theories of overeducation can be classified into these two camps. The trap perspective include career/job mobility theory (Galor and Sicherman, 1990), as well as signaling theory and screening theory (Aina and Pastore, 2020; Spence, 1973). It posits that overeducation functions as a source of scarring, stigma, or trapping effects for workers, suggesting a perceived lack of skills and leading to income penalties relative to adequately matched peers (Osseiran, 2020; Baert and Verhaest, 2019). The trade-off perspective include human capital theory (Becker, 1964), job competition model (Thurow, 1975), assignment model (Sattinger, 1993), adjustment theory (Baktash, 2025) and the job shopping theory (Jovanovic, 1979; Huertas and Raymond, 2024). They share the view that the additional human capital of overeducated workers is rewarded (Broniatowska, 2021; Johnes, 2019), implying greater competitiveness and the strategic use of overeducation to maximize earnings. Thus, they frame overeducation as an investment or a strategic trade-off for future income growth.

This study aims to test which of these theoretical paradigms best explains the consequences of overeducation in the pre- and post-expansion contexts at both the individual and group levels. At the individual level, whether overeducation leads to an income return (*a trade off*) or an income penalty (*a trap*) remains a subject of extensive debate, with numerous studies yielding inconsistent and inconclusive findings to date (Budria, 2011; Budria and Moro-Egido, 2008; Doon and Scicchitano, 2025; Tang and Wang, 2021). A core question is whether this effect has shifted due to higher education expansion according to the incomplete markets theory (Didier, 2024): was overeducation more likely a trade off in the pre-expansion cohorts, while it has transformed into an income trap in the post-expansion cohorts?

Another consequence of higher education expansion is social inequality. Although higher education expansion aims to reduce social inequality and promote an education-for-all society, some studies have found that the expansion has increased/maintained social inequality (Carvalhaes et al., 2023; Guo and Chen, 2023; Zhao, 2023), while others have found that it has reduced social inequality (Liu et al., 2016). Moreover, whether higher education expansion increases social inequality through overeducation remains unclear. Consequently, at the group level, a critical and unresolved question is whether overeducation acts as a key mechanism through which expansion influences inequality. In other words, within the new educational landscape, does overeducation amplify or mitigate existing disparities across gender, rural–urban, and socioeconomic lines? Is it a *trap* that disproportionately penalizes disadvantaged groups, thereby widening gaps, or could it function as a *trade-off* more accessible to the disadvantaged, potentially narrowing gaps?

To adjudicate between these competing theoretical propositions—trap versus trade-off—at both individual and group levels, this study employs a cohort design, which allow us to compare the differences in overeducation across cohorts that entered the labor market before and after the expansion, so as to isolate the effect of the higher education policy more clearly while controlling for other cohort-specific factors. Furthermore, as Broniatowska (2021) argues, the cohort approach enables researchers to investigate whether overeducation represents a persistent structural issue rather than a temporary mismatch, contributing to a more nuanced understanding of its causes and dynamics. A few cohort studies on overeducation have been conducted previously (Artz and Welsch, 2021; Baran, 2025; Bar-Haim et al., 2019; Xu and Xu, 2025), but they have often failed to address the collinearity between age, period, and cohort effects. To the best of our knowledge, this study is the first attempt in China to employ the Hierarchical Age-Period-Cohort model (HAPC) to examine the cohort-specific effects on overeducation and its associated income returns after resolving the collinearity problem.

We selected China as a critical case because its rapid, state-led higher education expansion provides a powerful natural experiment to observe how a supply-side shock reconfigures the labor market meaning of education. By employing a rigorous cohort design and the HAPC model to disentangle age, period, and cohort effects, this study aims to answer three questions: *(1) How did the incidence of overeducation shift across expansion cohorts? (2) At the individual level, did the individual income return to overeducation change from a trade-off to a trap? (3) At the group level, did the group disparities in income return to overeducation change, thereby exacerbating or alleviating social income inequality?* This approach moves beyond documenting the incidence of overeducation to test how its very consequence—as either a trap or a trade-off—is transformed at both the individual and group levels. The findings thus offer a nuanced resolution to longstanding theoretical debates and provide insights into the inequality dynamics of mass higher education relevant to many other developing countries.

The remainder of this paper is structured as follows. Section 2 reviews the previous literature and introduces a theoretical framework of trap versus trade-off alongside research hypotheses. Section 3 describes the data, cohort design, and the HAPC method. Section 4 presents the empirical results. Finally, Section 5 discusses the findings, implications, and limitations.

## 2 Literature review

### 2.1 Educational mismatch and overeducation

In the labor market, the demand for human capital and the supply of workers’ qualifications are not always perfectly matched (Zhang et al., 2023). Scholars generally regard educational attainment as a proxy for human capital and define the above phenomenon as the educational mismatch of the labor force (Duncan and Hoffman, 1981). There are two kinds of mismatch: vertical educational mismatch refers to the mismatch between graduates’ level of education and the requirement for their jobs, while horizontal mismatch refers to a poor match between the content of the graduates’ job and their field of education (Moore and Rosenbloom, 2016; Passaretta et al., 2023; Robst, 2007). Therefore, overeducation is a type of vertical mismatch in which a worker’s educational attainment exceeds the job requirements (Zhang et al., 2023).

The concept of overeducation was first proposed by Eckaus (1964), Berg (1970) and Freeman (1976), who focused on the difference in average education levels between the supply and demand sides (Zheng et al., 2021). It relates to the term under-employment, which focuses on the failure of the labor market to supply job-match positions, while overeducation describes a failure of the market for education to ensure that a position matches the educational background (Johnes, 2019). Therefore, we use the term “overeducation” to describe this phenomenon, as one consequence of the higher education expansion is the elevation of educational attainment levels, which may exceed the requirements of the available job market.

Measurements of overeducation include job analysis as well as subjective and objective measurements (Zhang et al., 2023). In the job analysis, experts specified the educational requirements for each occupation. However, as there are no authoritative assessment criteria in China thus far, they are not applicable. Subjective measurements asked the participants to evaluate whether their educational attainment matched their current occupation. Given the estimation bias caused by the respondents’ vanity or subjective concealment, this was not used in this study. Objective measurements were also called realized matches (Zhang et al., 2023), which calculate the average educational level of all practitioners within the occupation to estimate the education required by the job, comprising the mean and mode methods. The magnitude and significance of overeducation diverge among these measures (McGuinness, 2006; Rubb, 2003; Verhaest and Omey, 2006). Nonetheless, there is no consensus on the preferred indicator (Capsada-Munsech, 2019). Thus, the use of more than one indicator in these studies is advised (Capsada-Munsech, 2019). We used the mean method to calculate overeducation and the mode method as a robustness test.

The determinants of overeducation operate at distinct levels. At the macro level, workers may be pushed into positions that mismatch their education due to a confluence of factors, including mass higher education expansion, fluctuations in national human capital accumulation, labor demand, and competitiveness caused by economic recession or birth rate increase (Blázquez Cuesta et al., 2025; Groot and van den Brink, 2000; Summerfield and Theodossiou, 2017). Among them, higher education expansion represents a deliberate, large-scale, and enduring policy intervention that fundamentally alters the structure of labor supply. Unlike transient economic shocks, it systematically redefines educational credentials’ value and graduates’ lifelong career trajectories, making its analysis critical for understanding long-term societal shifts. However, as its effect interacts with other macroeconomic conditions, isolating the specific impact of expansion requires both statistically controlling for concurrent factors and adopting a design that captures its structural nature. This study therefore employs a cohort comparison between those entering the labor market before and after the major expansion, a design that is uniquely suited to distinguish the policy’s enduring intervention from transient period-specific fluctuations.

At the micro or group level, the risk and consequence of overeducation are not uniformly distributed. Significant disparities exist across groups defined by sex, age, area, family background, and other attributes (Burris, 1983; Capsada-Munsech, 2020). These attributes shape access to information, networks, and types of human capital, leading to varied vulnerability to mismatches and divergent capacities to navigate them. Thus, examining overeducation without considering group heterogeneity offers an incomplete picture. Analyzing how the experience of overeducation differs across these groups—and, crucially, how these differentials may have been altered by the macro-level shock of expansion—is vital for understanding whether mass higher education mitigates or reproduces existing social inequalities.

### 2.2 Overeducation and income returns: between a ‘trap’ and a ‘trade-off’

A central debate in the literature concerns the socioeconomic consequence of overeducation (Burris, 1983; Ma et al., 2022; Montuenga and García-Mainar, 2025; Wu et al., 2024; Zhang et al., 2023), particularly its impact on income, with empirical studies reporting contradictory findings of both positive and negative returns. For example, some studies in Trinidad and Tobago, Poland, Germany, Ghana, Kenya, the United States, China, and other G7 countries have found that overeducation has a positive effect on income (Baran, 2024; Broniatowska, 2021; Carmichael et al., 2021; Doon and Scicchitano, 2025; Habibi and Kamis, 2021; Johnes, 2019; Kamis and Habibi, 2022; Xiangrong, 2008). Other studies have found that overeducation reduces income (De Santis et al., 2022; Eguia et al., 2023; Elamin, 2023; Li et al., 2015; Palczyńska, 2021; Sun and Kim, 2022). This inconsistency stems not only from methodological differences but, more fundamentally, from the divergent theoretical assumptions underpinning the phenomenon. These theories can be synthesized into two competing paradigms regarding the nature and consequence of overeducation: the ‘trade-off’ and the ‘trap’.

The ‘trade-off’ perspective posits that overeducation can be a strategic choice with compensatory benefits. For example, *human capital theory* (Becker, 1964) assumes that individuals invest in education to use it in the labor market and maximize their utility and wages, while firms are willing to fully utilize workers’ skills and knowledge to maximize their productivity. According to this theory, According to this theory, an adequately educated or undereducated worker may be outcompeted or subject to the *crowding out effect* by an overeducated worker, resulting in an income return from overeducation (Habibi and Kamis, 2021; Kamis and Habibi, 2022).

Similarly, *the job competition model* (Thurow, 1975) suggests that workers’ positions in the queue of the labor market depend on their education level relative to the rest of the workers. Thus, individuals with more education obtain the best jobs. However, even workers in the highest positions may be overeducated if there are no jobs left in the queue that match their education level. The difference between the two theories is that human capital theory suggests that overeducation is temporary, while job competitive model suggests that overeducation can become a permanent state if no new high-skilled jobs are on offer (Huertas and Raymond, 2024; Kiersztyn, 2013).

Situated between human capital theory and the competitive job model, the *assignment model* (Sattinger, 1993) stresses that workers’ and firms’ characteristics are instrumental in allocating individuals to jobs. First, individuals choose a sector based on their wage maximization preferences. After this intermediate step, overeducation can be resolved through individual or firm adjustments. This theory is similar to the *adjustment theory* (Baktash, 2025) or the *step-stone effect* (Albert et al., 2023) that assumes that overeducated workers sort into performance pay jobs as an adjustment mechanism or a stepstone that performance pay enhances their wages.

Further supporting the trade-off view, *job shopping theory* assumes that overeducation may be a strategic asset: individuals may accept less-demanding jobs than the ones that they are capable of performing as a way to enter jobs commensurate with their education level (Jovanovic, 1979; Huertas and Raymond, 2024). Individuals may temporarily accept jobs below their education level as a means to enter a desirable firm, industry, or location, using it as a strategic foothold for future advancement into roles that ultimately match their educational qualifications.

In conclusion, these theories share the view that the additional human capital of overeducated workers is rewarded (Broniatowska, 2021; Johnes, 2019), implying greater competitiveness and the strategic use of overeducation to maximize earnings. Thus, they frame overeducation as an investment or a strategic *trade-off* for future income growth (Johnes, 2019).

In contrast, the ‘trap’ perspective conceptualizes overeducation as a suboptimal and often persistent state that signals a deficiency and incurs penalties. This view is anchored in theories that emphasize individual constraints and market inefficiencies. For example, *career/job mobility theory* (Galor and Sicherman, 1990) argues that workers become overeducated because they cannot clearly signal their knowledge and skills, or they are lacking work experience and/or work-specific skills. This aligns with the *signaling theory and screening theory*, which posit that graduation beyond the minimum period entails a negative signal to employers: longer delays hint at unobserved characteristics with a negative productivity value (Aina and Pastore, 2020; Spence, 1973). Overeducation thus functions as a source of *scarring, stigma, or trapping effects* for workers, suggesting a perceived lack of skills and leading to income penalties relative to adequately matched peers (Osseiran, 2020; Baert and Verhaest, 2019). A number of studies also supported these ‘trap’ perspective theoretical mechanisms and have documented such negative effects in practice (Acosta-Ballesteros et al., 2018; Alba-Ramirez, 1993; Albert et al., 2023; Eguia et al., 2023; Esposito and Scicchitano, 2022).

This theoretical dichotomy—whether overeducation functions as a *trade-off* or a *trap*—provides a crucial framework for reconciling mixed empirical evidence. It also raises a pivotal, context-dependent question: under what conditions, such as before or after a massive higher education expansion, does one dynamic dominate the other?

The *incomplete markets theory* provides a dynamic model for understanding how the income returns to overeducation can shift over time, particularly following a supply shock like mass higher education expansion (Didier, 2024). This process will lead to a three-moment scenario. In the first moment, assimilated to incomplete market behavior, the increased demand for more educated workers (occupational upgrading) leads to wage premiums associated with overeducation. This aligns with the ‘trade-off’ perspective. In a second moment, the labor market updates its educational preferences, clarifying its expectations about the equivalence of educational attainment to some productivity levels for a given occupation. In this stage, overeducation does not produce wage premiums or penalties by advancing to an optimal job sorting of workers’ educational attainment to occupations. That would be expected in a mature or complete market, where the occupational structure of the labor market remains stable. Finally, given the inorganic coevolution of educational and labor markets, some sections of the labor market hire people with more significant human capital accumulation than required by the occupation, detaching the process of occupational upgrading of the market from the skill-upgrading of the workforce. In this scenario, the workforce copes with wage penalties associated with overeducation, a stagnation of higher education returns, and significant problems in getting an appropriate job for their educational profile (underemployment). In this scenario, the incomplete market ceases to function as a temporary arena for individual trade-offs and instead institutionalizes a systemic ‘trap’, leading to widespread wage penalties, stagnating returns to education, and underemployment.

This three-stage model offers a powerful theoretical basis for our hypothesis: China’s rapid, state-led higher education expansion may have dramatically accelerated the transition through these stages. It posits that the pre-expansion cohort likely experienced the first or early second stage (where overeducation could function as a trade-off), while the post-expansion cohort entered a labor market already in or quickly reaching the third stage, where overeducation operates primarily as a structural trap. This framework directly informs our investigation into whether the income effect of overeducation has fundamentally transformed from a positive or neutral return to a negative one across the expansion divide.

### 2.3 Group disparities: heterogeneous effects and inequality implications

The debate between ‘trap’ and ‘trade-off’ gains further complexity and social significance when examined through the lens of group disparities. The expansion of higher education, while aims to reduce inequality (Araki, 2023; Wu and Zheng, 2008), has had inconsistent effects on social inequality. For instance, 26% of China’s top universities, numbering approximately 100 institutions, and 30% of central government-managed universities are located in Beijing, where the gross higher education enrollment rate exceeds 50%, which is more than double the national average. Research in Brazil also found that enrollment more than doubled in 14 years, but growth among the top 1% of institutions was higher than the total growth among the bottom 90% of institutions (Carvalhaes et al., 2023).

However, some results did not support the hypothesis that inequality was enlarged or maintained (Liu et al., 2016). Studies on China’s higher education expansion in 1999 found that the expansion interrupted the continuously enlarged gap of access opportunities between regions, narrowed rural inequality within provinces, and reduced the gender gap and inequalities affected by household registration, family economic status, and parental education level (Cin et al., 2021; Rong and Deng, 2022; Wu et al., 2020). Nevertheless, Aldea and Zamfir (2025) conducted a bibliometric analysis and found that the expansion of education positively influenced educational inequalities, especially gender inequalities.

This inconsistency extends to the role of overeducation in shaping social inequality (Budria, 2011; Huertas and Raymond, 2024). For example, some studies found that overeducation contributed to group dispersion (Budria and Moro-Egido, 2008; Tang and Wang, 2021); while Doon and Scicchitano (2025) found that overeducation reduced income inequality, and Budria (2011) found that the positive effect of education on wage inequality is not due to the prevalence of educational mismatches in the labor market.

Apparently, the income consequences of overeducation are unlikely to be uniform across all groups. The two theoretical paradigms outlined above imply heterogeneous effects when applied to a stratified society. The ‘trade-off’ perspective suggests that overeducation may be particularly significant for disadvantaged groups. For individuals lacking superior social networks or financial safety nets, accepting an initially mismatched job can serve as a critical strategy to gain a foothold in competitive sectors or locations, thereby reducing the income gap with more advantaged peers. Conversely, the ‘trap’ perspective suggests that overeducation may result in more severe consequences for disadvantaged groups. The lack of resources to negotiate wages, transition to better-matched jobs, or counteract negative signaling can render an initial overeducation more persistent and damaging for disadvantaged groups, exacerbating existing social inequalities.

Consequently, whether overeducation ultimately reduces or reproduces income inequality hinges on which paradigm predominates for different segments of the population. If overeducation acts primarily as a trap that disproportionately penalizes disadvantaged groups, it will widen income inequality. If, however, it functions more as a trade-off that offers greater relative returns to disadvantaged groups, it could narrow disparities. Therefore, analyzing average effects alone is insufficient; a complete understanding requires examining the heterogeneous returns to overeducation across key social groups. This study directly addresses this by investigating how the ‘trap vs. trade-off’ dynamic varies by gender, rural–urban origin, and family socioeconomic status, thereby clarifying the mechanism through which educational expansion influences social inequality.

### 2.4 Theoretical framework and hypotheses

This study aims to systematically examine how China’s mass higher education expansion has reshaped the nature and socioeconomic consequences of overeducation. Moving beyond previous research that primarily described its incidence or average income effects, this study employs a cohort design to focus on the structural shifts induced by this pivotal policy shock. Theoretically, it is framed by the ‘trap versus trade-off’ dichotomy. At the individual level, the study examines whether the income effect of overeducation has fundamentally shifted from a trade-off to a trap following the higher education expansion. At the group level, it investigates whether overeducation functions as a trap that exacerbates or a trade-off that mitigates income inequality across gender, rural–urban, and family background. Methodologically, the study advances the field by employing a Hierarchical Age-Period-Cohort (HAPC) model to better disentangle age, period, and cohort effects and isolate the long-term cohort effects of the expansion from the confounding influences of other macro-factors. To our knowledge, this study is the first attempt in China to employ HAPC to examine the cohort-specific effects on overeducation and its associated income returns.

Based on the theoretical framework of the ‘trap versus trade-off’ dichotomy and the analysis of China’s expansion policy context, this study proposes the following series of hypotheses to guide subsequent empirical testing. While the literature generally posits that expansion leads to increased overeducation, most evidence relies on cross-sectional comparisons or theoretical deductions, lacking rigorous testing based on long-term cohorts. This study provides the first cohort-based verification:

*H1*: The incidence of overeducation is significantly higher among cohorts entering the labor market after the expansion compared to cohorts entering before it.

Moreover, the groups most affected by higher education expansion remain unclear. We proposed two opposing hypotheses regarding group disparity. H2a assumes that disadvantaged groups (e.g., female or rural residents) have a higher level of overeducation than privileged groups (e.g., male or urban residents), whereas H2b assumes the opposite:

*H2a*: After the expansion, disadvantaged groups (e.g., females, rural residents) exhibit a greater increase in the incidence of overeducation compared to advantaged groups.

*H2b*: After the expansion, advantaged groups (e.g., males, urban residents) exhibit a greater increase in the incidence of overeducation compared to disadvantaged groups.

Empirical findings on the income returns to overeducation are profoundly inconsistent. Grounded in the core theoretical dichotomy and informed by the dynamic view of incomplete markets theory—which posits a shift from wage premiums to penalties as the labor market adapts to a supply shock—this study directly tests the proposition that the expansion has fundamentally altered its nature:

*H3*: The income effect of overeducation has transformed from a ‘trade-off’ to a ‘trap’ after the expansion, with pre-expansion cohorts experiencing greater income returns from overeducation, while post-expansion cohorts facing more income penalties.

A critical yet unresolved question is whether overeducation mitigates or reinforces pre-existing social inequalities through differential income effects. We formulate two opposing hypotheses:

*H4a (trap-driven inequality)*: After the expansion, the income penalty associated with overeducation is more severe for disadvantaged groups than for advantaged groups, thereby widening inter-group income inequality.

*H4b (trade-off-driven convergence)*: After the expansion, the income return associated with overeducation is greater for disadvantaged groups than for advantaged groups, thereby narrowing inter-group income inequality.

## 3 Methods

### 3.1 Data and participants

Data were sourced from the Chinese General Social Survey (CGSS), a comprehensive and nationally representative social survey project launched in 2003 and conducted by Renmin University of China^1^. From its inception until 2008, the CGSS primarily surveyed urban residents. Starting in 2010, it transitioned to a full nationally representative sample covering both urban and rural populations. It employs a multi-stage, stratified probability-proportional-to-size (PPS) sampling method to ensure representativeness at both the national and regional levels. The CGSS is recognized as the earliest longitude national representative social survey in the Chinese Mainland, which covers a wide range of socioeconomic topics, including education, employment, income, and family background, making it particularly suitable for studying educational mismatch and labor market outcomes. Its longitudinal design allows for robust cohort-based analyses of policy impacts over time. The CGSS is widely used in social science research and has been extensively validated for its reliability and academic credibility.

Based on the evolution of the CGSS sampling framework, this study utilizes data from the 2010, 2011, 2012, 2013, 2015, 2017, 2018, and 2021 waves to construct a multi-year cross-sectional dataset. This selection is made to ensure the sample consistently covers both urban and rural populations. As macroeconomic data for the control variables have only been available since 1949, the study only recruited participants born after 1949. After logic tests and missing data were eliminated, there were 50,932 participants, aged 17–60 years and born between 1950 and 2003. Among all participants, 26,566 (52.16%) were female, and 19,166 (37.63%) were from rural areas. Descriptions of the variables are presented in Table 1.

**Table 1:** Variables.

Variables	Coding	Mean/percentage
Cohorts	1 ~ 19	8.82 ± 3.96
Overeducation – mean	−15.01 ~ 12.98	−0.05 ± 3.34
Overeducation – mode	−16 ~ 13	−0.30 ± 3.66
Log of income	0 ~ 16.12	7.55 ± 4.30
Sex: female	=0	52.16%
Sex: male	=1	47.84%
Rural residents	=0	37.63%
Urban residents	=1	62.37%
Region: eastern	=1	40.69%
Region: central	=2	37.81%
Region: western	=3	21.50%
Parents’ occupations: managers	=1	7.98%
Parents’ occupations: professionals/technicians	=2	9.08%
Parents’ occupations: clerks/servicers	=3	8.65%
Parents’ occupations: workers/farmers	=4	74.29%
Individual occupations: never worked full-timely	=0	5.38%
Individual occupations: managers	=1	8.11%
Individual occupations: professionals	=2	7.04%
Individual occupations: technicians	=3	6.56%
Individual occupations: clerks	=4	7.48%
Individual occupations: servicers	=5	13.31%
Individual occupations: skilled workers/farmers	=6	0.75%
Individual occupations: craft workers	=7	12.87%
Individual occupations: operators	=8	7.94%
Individual occupations: elementary occupations	=9	30.55%
Age	17 ~ 60	41.94 ± 11.44
Han Chinese: no	=0	8.28%
Han Chinese: yes	=1	91.72%
Members of the Communist Party of China: no	=0	90.48%
Members of the Communist Party of China: yes	=1	9.52%
Religion: no	=0	89.30%
Religion: yes	=1	10.70%
Hukou status: rural	=1	56.95%
Hukou status: urban	=2	30.59%
Hukou status: residential	=3	12.46%
Subjective health status	1 ~ 5	3.71 ± 1.06
Parent’s education: elementary	=1	62.49%
Parent’s education: junior secondary	=2	19.85%
Parent’s education: high secondary	=3	12.99%
Parent’s education: tertiary	=4	4.67%
Subjective socioeconomic status	1 ~ 10	4.21 ± 1.69
Subjective socioeconomic status when aged 14	1 ~ 10	3.24 ± 1.85
Log of GDP per capita at birth	4.80 ~ 9.31	5.97 ± 0.92
GDP growth per capita at birth	89 ~ 113.1	105.85 ± 5.40
Birth rate at birth	12.52 ~ 40.31	27.71 ± 7.42
Enrollment rate at 17 to 19	0.04 ~ 1.25	0.37 ± 0.29

### 3.2 Measures

#### 3.2.1 Cohort

As the expansion of higher education in China began in 1999, this study used 1999 as the cutoff point. Cohorts were defined by three-year intervals before or after 1999, yielding a total of 19 cohorts for analysis.

#### 3.2.2 Overeducation

Normally, individuals with education exceeding the average years of education within the occupation by more than one standard deviation in each survey year are considered overeducated (Geißler, 2025). However, this study treats overeducation as a continuous rather than a categorical variable, quantifying it as the difference in years between an individual’s education and the average for their job. A positive value indicates overeducation, and a negative value denotes undereducation. The education required by the job can be measured by realized matches, which calculate the average educational level of all practitioners within the occupation using the mean and mode methods (Zhang et al., 2023). The former uses the mean as a criterion, whereas the latter uses the mode as a criterion. In this study, we used the mean method to perform the baseline analysis and the mode method to conduct the robustness test.

In the CGSS, the participants’ educational level was measured and then transferred to the educational level. The CGSS measured the participants’ current or former non-agricultural occupations according to the 1988 or 2008 version of the International Standard Classification of Occupations (ISCO-88 or ISCO-08). To ensure consistency, the original occupational codes based on the ISCO-08 were converted to the ISCO-88 classification. The ISCO-88 comprises four levels: major (10), submajor (28), minor (116), and unit (390). In this study, we used submajor categories as the basis for classification. Farmers were coded into subgroups of agricultural, fishery, and related laborers. Participants who never worked full-timely were assigned to a new subgroup. We did not treat these participants as missing variables because we also aimed to examine whether, with the expansion of higher education, more highly educated individuals (with more years of schooling) could not find employment.

#### 3.2.3 Income

This study used annual occupational income to examine income returns from overeducation. In the CGSS, annual occupational income was assessed using the question, “What was your total occupational/labor income for the last year?.” The log of income was used in the analysis to improve the model fit and allow for a more intuitive interpretation of the results as percentage changes.

#### 3.2.4 Group variables

The study also analyzed disparities across different demographic groups, including differences in region, sex, family background, and occupation. This region was captured using two distinct variables. Given China’s significant rural–urban and regional disparities, this study first classified the regions into rural (=0) and urban (=1). Second, the regions were categorized into eastern (1), central (2), and western (3) areas. Sex was a binary variable divided into female (=0) and male (=1). Family background was indicated by the parents’ occupations. Although many studies have chosen fathers’ occupations as the primary indication, recognizing the growing number of mothers who are active in the workforce and attain higher professional positions than fathers, we selected the highest occupational status between the two parents as an indicator of family background. The parents’ occupations were categorized as managers (1), professionals/technicians (2), clerks/servicers (3), and workers/farmers (4). To narrow down the number of occupations, individual occupations were categorized using the major categories of ISCO-88; armed forces (*N* = 7) were merged into managers, considering their high reputation and prestige in China. Finally, occupation includes ten categories: never worked full-timely (=0), managers (=1), professionals (=2), technicians (=3), clerks (=4), servicers (=5), skilled workers/farmers (=6), craft workers (=7), operators (=8), and elementary occupations (=9).

#### 3.2.5 Individual control variables

Individual control variables included group variables not used as independent variables in the analysis: age and age squared, Han Chinese or ethnic minorities, religion, members of the Communist Party of China, Hukou status, marital status, subjective health status, highest educational level of parents, subjective socioeconomic status, and subjective socioeconomic status when aged 14.

#### 3.2.6 Macro socioeconomic control variables

The macro-socioeconomic control variables included those that might influence cohort differences in education, occupation, and income. These are the average log of GDP per capita from 1 year before birth to 1 year after birth, average GDP growth per capita from 1 year before birth to 1 year after birth, average birth rate from 1 year before birth to 1 year after birth, and enrollment rate of higher education when age reached 17–19. Using the average values across the 3 years helps better control for the impact of the preceding and subsequent birth cohorts on individuals.

### 3.3 Analytic strategy

This study employs a dual analytical focus to investigate the consequences of China’s higher education expansion. First, it examines differences in the incidence of overeducation across birth cohorts entering the labor market before and after the expansion, assessing whether the policy exacerbated overeducation and its distribution among social groups. Second, by comparing the effect of overeducation on income across these cohort groups, it evaluates whether the income return to overeducation has transformed, thereby assessing its role as either a ‘trap’ or a ‘trade-off’ at both individual and group levels. Therefore, in the first part, the dependent variable is an individual’s overeducation status (overeducation); while in the second part, the dependent variable is the individual’s income level (income). However, to clearly present the effect of overeducation on income, the regression coefficient of overeducation on income is reported as the primary estimand of interest in the second part of the analysis.

Moreover, when analyzing generational differences, age and period effects may confound the results. Owing to the colinear relationship among age, period, and cohort, it is impossible to obtain a unique solution for the model parameters, leading to the “identification problem” in the age-period-cohort (APC) model (Fienberg and Mason, 1978). To address the “identification” issue of the APC model, the Hierarchical Age-Period-Cohort (HAPC) model effectively resolves the collinearity problem among the three factors (Yang, 2008). The HAPC model is essentially a hierarchical model that groups birth years into cohorts—each cohort has at least two birth years. This allows age to be nested within birth cohorts and survey years, treating survey years and cohorts as second-level variables, while age is treated as a first-level variable, breaking the collinear relationship among the three and making the model “identifiable.”

Though there are other methods to address the APC identification problem, including the Intrinsic Estimator (APC-IE) and the Age-Period-Cohort-Interaction (APC-I) model. However, the HAPC was selected for this study due to its superior alignment with our research questions and data structure, offering distinct advantages for our analysis. First, the HAPC does not require the strict assumption of equidistant age and period intervals, which our data cannot satisfy. More critically, its multilevel, cross-classified structure provides a key advantage: it allows us to not only estimate overall APC trends but also to examine how period and cohort effects vary across social groups (e.g., by urban–rural status or sex). Alternative APC models are designed to decompose aggregate trends and lack this capacity to model contextual heterogeneity. Therefore, the HAPC is uniquely suited to test our core hypotheses, as it enables a direct investigation of whether the expansion’s legacy operates uniformly or serves as a divergent trap/trade-off across different segments of society. The model specifications are as follows:

Level 1 model:

*overeducation_ijk_/income_ijk_* = *β*_0*jk*_ + *β*_1_*AGE_ijk_* + *β*_2_*AGE^2^_ijk_* +*β*_3_*X_ijk_* + *e_ijk_, e_ijk_* ∼ *N* (0*,σ*^2^) (1)

Level 2 model:

*β*_0*jk*_ = *γ*_0_ + *u*_0*j*_ + *v*_0*k*_ + *u*_m*j*_ + *v*_n*k*_*, u*_0*j*_ ∼ *N* (0*,τ_u_*)*, v*_0*k*_ ∼ *N* (0*,τ_v_*) (2)

Overall Model:

*overeducation_ijk_/income_ijk_* = *γ*_0_ + *β*_1_*AGE_ijk_* + *β*_2_*AGE^2^_ijk_* + *β*_3_*X_ijk_* + *u*_m*j*_ + *v*_n*k*_ + *u*_0*j*_+ *v*_0*k*_ + *e_ijk_* (3)

Among them, *β*_0*jk*_ represents the average score of overeducation/income for the j-th period and the k-th cohort; *β*_1_ is the age coefficient; *β*_2_ is the age squared coefficient; *β*_3_ represents the fixed coefficients of other control variables at level one; *X* denotes the independent variables*; u*_m*j*_ and *v*_n*k*_ represent the macro variables for the period and cohort, respectively; *e_ijk_* is the random error at the individual level, indicating the difference between individual *ijk* and the average of their group, assumed to follow a normal distribution with a mean of 0 and a variance of *σ*^2^; *γ*_0_ represents the overall intercept, indicating the total average value when the random effects of the period and cohort are at their means and the other independent variables are 0, reflecting the overall average score of overeducation/income; *u*_0*j*_ is the random effect for period *j* when the cohort effect is at its mean, assumed to follow a normal distribution with a mean of 0 and a variance of *τ_u_*; *v*_0*k*_ represents the random effect for cohort *k* when the period effect is at its mean, assumed to follow a normal distribution with a mean of 0 and a variance of *τ_v_*; *u*_0_ + *γ*_0_ indicates the trend of variation in overeducation/income over time; while *v*_0_ + *γ*_0_ represents the differences in overeducation/income across different cohorts.

For analyzing the incidence of overeducation (H1 and H2), the dependent variable is overeducation status; to test the group differences in the incidence of overeducation (H2), a cohort × group interaction term was introduced. For analyzing the income return (H3 and H4), the dependent variable switches to log(income). To test the shift from trade-off to trap (H3), we include an overeducation × cohort interaction term. To test group-level inequality (H4), we include an overeducation × cohort × group triple interaction term.

## 4 Results

### 4.1 Overeducation across cohorts

The analysis primarily focuses on variation across cohort groups; accordingly, the results presented concentrate on the estimates of the random effects to maintain a concise presentation. In the base model (Table 2, Model 1), we analyzed the cohort effect of overeducation without the control variables. The results showed a significant cohort variance (*B* = 0.15, SE = 0.05, *p* = 0.004). After individual control variables and macro socioeconomic variables were controlled (Table 2, model 2 and model 3), cohort variance was significant (*Bs* = 0.08–0.15, SE = 0.03–0.05, *ps* = 0.004–0.009), indicating significant cohort differences in overeducation.

**Table 2:** Random effects of overeducation.

Variance	Model 1	Model 2	Model 3	Model 4	Model 5	Model 6	Model 7	Model 8
Period	0.04^*^ (0.02)	0.03^*^ (0.02)	0.01 (0.01)	0.01 (0.01)	0.01 (0.01)	0.01 (0.01)	0.01 (0.01)	0.03 (0.02)
Cohort	0.15^**^ (0.05)	0.15^**^ (0.05)	0.08^**^ (0.03)	0.09^**^ (0.04)	0.13^**^ (0.06)	0.06^*^ (0.04)	0.04^*^ (0.02)	0.01 (0.02)
Gender × cohort				0.19^**^ (0.08)				
Rural–urban × cohort					0.11^**^ (0.04)			
Living area × cohort						0.06^***^ (0.02)		
Parents’ occupation × cohort							0.02^**^ (0.01)	
Individual occupation × cohort								0.36^***^ (0.05)
Residual	10.35^***^ (0.06)	9.17^***^ (0.06)	9.17^***^ (0.06)	9.14^***^ (0.06)	9.15^***^ (0.06)	9.14^***^ (0.06)	9.16^***^ (0.06)	8.05^***^ (0.05)
BIC	263656.4	257593.5	257584.8	257477.3	257484.3	257465.0	257567.9	247724.3

Model 1 was the model without control variables; model 2 was controlled for individual variables; model 3 ~ 8 were controlled for individual and macro socioeconomic variables. ^*^ *p* < 0.05; ^**^ *p* < 0.01; ^***^ *p* < 0.001.

**Figure 2:**
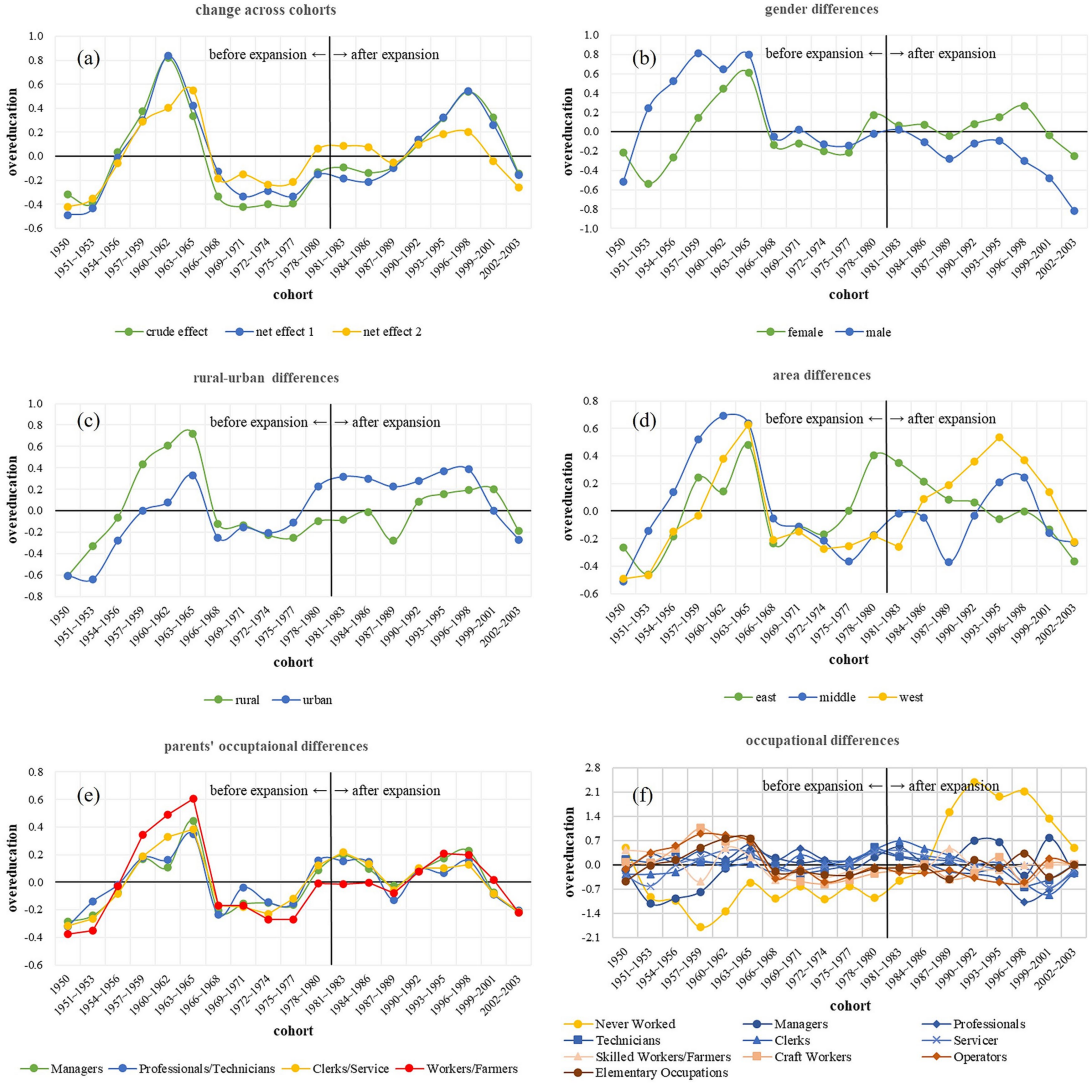
Overeducation across cohorts. Panel **(a)** shows the change of overeducation before and after expansion. Panels **(b-f)** shows changes of overeducation before and after expansion among gender, rural-urban groups, areas, parents’ occupations and individual occupations differences. Y axis is year of overeducation, positive means overeducation, negative means undereducation. In **(a)**, crude effect is the estimated change of overeducation without controlling variables; net effect 1 is the estimated change of overeducation when individual variables controlled; net effect 2 is the estimated change of overeducation when all the individual and macro socioeconomic variables were controlled.

Figure 2A shows the results of random effects. Regardless of the crude effect without control variables or net effects controlled for individual and/or macro socioeconomic variables, the cohort effect of overeducation showed two peaks. The first peak occurred between 1960 and 1965 cohorts. People born between 1960 and 1965 in China were right at the intersection of the “Sent-Down Youth” movement and the restoration of the college entrance exams. The overeducation peak observed within this cohort can be attributed to the socio-historical conditions of the period, which were shaped by policies that had relocated intellectually advanced students to rural areas for agricultural modernization.

The second peak is around 1996–1998 cohorts; after higher education expansion, we can observe a clear increase in overeducation, especially in the crude effect and net effect 1, when only individual variables were controlled. However, the drop in overeducation after 1998 cohorts may have been due to the declining birth rate accompanying the expansion of higher education. Specifically, while the expansion of higher education increased competitive pressure in the job market, potentially leading to an increase in overeducation, the declining birth rate simultaneously reduced this pressure. With the continued decrease in birth rates, by the year 1998, the reduction in competitive pressure due to the lower birth rate had surpassed the increase caused by the expansion of higher education, resulting in a decline in overeducation. After controlling for the four macro-socioeconomic variables, the second peak in net effect 2 was lower than that in the crude effect and net effect 1. Thus, these macro-level factors (enrollment rate, birth rate, GDP per capita, and GDP growth) partially accounted for the emergence of the peak. Therefore, H1 is only partially supported.

### 4.2 Overeducation across cohorts by groups

We examined overeducation across the cohorts by groups. In Table 2 (model 4 to model 8), results showed that the interaction variance of cohort and sex, rural–urban regions, living areas, parents’ occupations and individual occupations were significant (*Bs* = 0.02–0.40, SE = 0.01–0.08, *ps* = 0.000–0.008)—there were significant sex, rural–urban, regional, family background and occupational disparities in overeducation among cohorts.

Specifically, Figure 2B shows that before expansion, males had more years of overeducation than females; nevertheless, after expansion, females had more years of overeducation than males. In other words, after expansion, to get the same job, females had to have higher education levels than males.

As shown in Figure 2C, before the expansion, rural residents had more years of overeducation than urban residents; however, after the restoration of college entrance exams, urban residents had more years of overeducation than rural residents. The differences increased after expansion, but among cohorts born after 1998, rural residents had more years of overeducation than urban residents. These findings highlight several key points in this study. First, prior to the restoration of the college entrance exams, the “Sent-Down Youth” movement contributed to a relatively higher overeducation among rural residents. Second, after the restoration of college entrance exams, especially following the expansion of higher education enrollment, urban residents had to have higher levels of education within the same industry, which may be attributed to competitive pressures in non-agricultural employment. Third, the recent reversal of this trend suggests reduced opportunities for rural residents, consistent with ongoing public discourse and research on reduced social mobility opportunities among rural residents (Xie et al., 2022).

As shown in Figure 2D, before the restoration of college entrance exams, people living in middle China had the highest level of overeducation, whereas after the restoration of college entrance exams, people living in east China had the highest level of overeducation. After the expansion of higher education enrollment, people living in western China had the highest level of overeducation. The results indicate that after the expansion, people living in western China had to have a higher level of education to obtain the same job.

As shown in Figure 2E, before the restoration of the college entrance exams, participants whose parents were farmers or workers had the highest level of overeducation, whereas after the restoration, they had the lowest level of overeducation, and participants whose parents were professionals or technicians had a higher level of overeducation. Therefore, this result proved our speculation above that the peak of overeducation around 1960–1965 cohorts may be because of the “Sent-Down Youth” movement and the restoration of the college entrance exams relocated intellectually advanced students to rural areas for agricultural modernization, resulting in overeducation of farmers or workers. However, after the expansion of higher education, differences in family background changed again, and the overeducation level of participants whose parents were farmers or workers turned out to be higher, whereas the overeducation level of participants whose parents were professionals or technicians turned out to be lower, indicating a return to social inequality, where lower family background participants had a higher level of overeducation.

As for individual occupations (Figure 2F), the most notable change was that after the expansion of higher education, participants who never worked had the highest level of overeducation. Before the restoration of the college entrance exams, participants who were lower in occupation had the higher level of overeducation. Nonetheless, after restoration and expansion, overeducation in lower occupations decreased, whereas overeducation in higher occupations increased. Nevertheless, among cohorts born after 1996–1998, overeducation had rebounded to relatively high levels in certain low-status occupations. Therefore, in terms of competition within different industries, after the expansion of higher education, overeducation in low-status occupations decreased, likely due to reduced competition within these occupations, whereas overeducation in high-status occupations increased, possibly because of intensified competition. However, a recent relative rise in overeducation has also been detected in some low-status occupations, which may be related to the overall increase in educational attainment and the back-to-homeland wave in which some university graduates choose to return to and contribute to their home villages (Xinhua, 2024).

In conclusion, the rural–urban and individual occupational differences supported H2b more, while differences related to sex, region, and family background indicated that the expansion of higher education enrollment widened social class disparities, supporting H2a. After the expansion, disadvantaged groups, such as females, people living in western China, and people whose parents were farmers/workers had to pursue more years of education to enter the same industry.

### 4.3 Income return of overeducation

**Figure 3:**
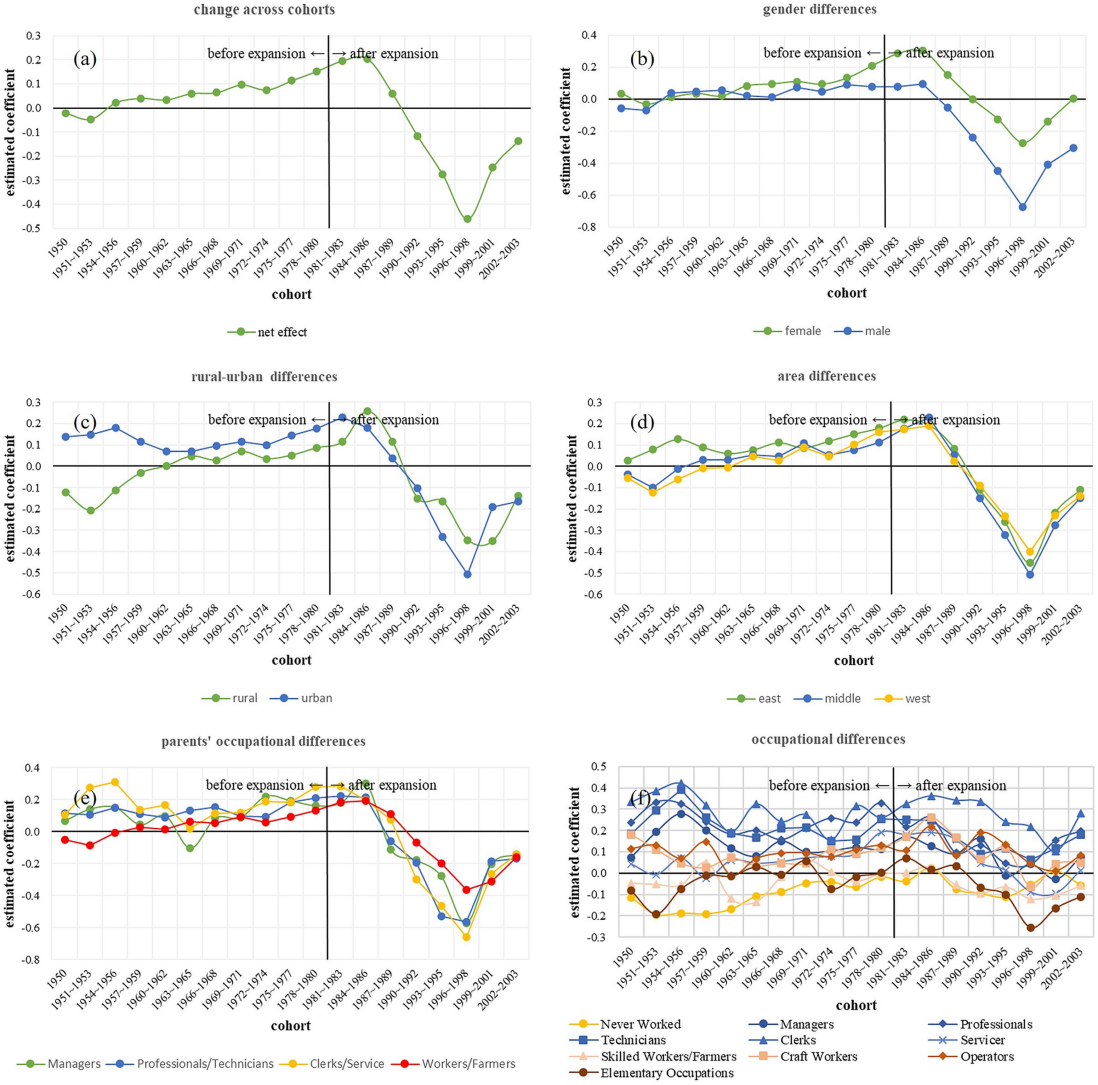
Estimated coefficients of overeducation on income across cohorts. Panel **(a)** shows the change of estimated coefficients of overeducation on income across cohorts. Panels **(b-f)** shows changes of estimated coefficients of overeducation on income across cohorts among gender, rural-urban groups, areas, parents’ occupations and individual occupations differences. Y axis is the estimated coefficients of overeducation on income; positive means income returns, negative means income penalty. Net effect in **(a)** is the estimated effect when all the individual and macro socioeconomic variables were controlled.

In Table 3 (Model 1), we examine the income returns from overeducation among different cohorts. The results showed significant cohort differences; overeducation by cohort variance was significant (*B* = 0.03, SE = 0.01, *p* = 0.003). Figure 3A shows that before the expansion, the income return from overeducation increased; however, for cohorts born after 1986, the income return from overeducation decreased, and for cohorts born in 1998, it increased again. Nevertheless, despite a subsequent increase, the coefficients indicated that after the expansion of higher education, the effect of overeducation on income became negative. Thus, before the expansion, overeducation was associated with an income return, whereas after the expansion, it resulted in an income penalty, showing a change from a tradeoff effect to a trapping effect. H3a was supported— overeducation leads to an income penalty after higher education expansion, although the associated income penalty diminishes among younger cohorts.

**Table 3:** Random effects of overeducation on income returns.

Variance	Model 1	Model 2	Model 3	Model 4	Model 5	Model 6
Period	0.08 (0.08)	0.08 (0.08)	0.08 (0.08)	0.08 (0.08)	0.08 (0.08)	0.25^*^ (0.14)
Cohort	1.05^**^ (0.45)	1.06^**^ (0.45)	1.06^**^ (0.45)	1.04^**^ (0.45)	1.07^**^ (0.45)	0.02 (0.01)
Overeducation × cohort	0.03^**^ (0.01)	0.02^**^ (0.01)	0.03^**^ (0.01)	0.03^**^ (0.01)	0.04^**^ (0.02)	0.002 (0.002)
Gender × overeducation × cohort		0.02^*^ (0.01)				
Rural–urban × overeducation × cohort			0.02^**^ (0.01)			
Living area × overeducation × cohort				0.003^*^ (0.001)		
Parents’ occupation × overeducation × cohort					0.01^***^ (0.003)	
Individual occupation × overeducation × cohort						0.01^***^ (0.002)
Residual	14.46^***^ (0.09)	14.41^***^ (0.09)	14.41^***^ (0.09)	14.44^***^ (0.09)	14.41^***^ (0.09)	12.45^***^ (0.08)
BIC	280870.2	280732.3	280,725	280842.2	280795.9	269462.2

Individual and macro socioeconomic variables were controlled. ^*^ *p* < 0.05; ^**^ *p* < 0.01; ^***^ *p* < 0.001.

### 4.4 Income return of overeducation by groups

There were significant income return differences by group (Table 3; Models 2–6). The three-way interaction variance between groups (sex, rural–urban regions, living areas, parents’ occupations, and individual occupations), overeducation, and cohort was significant (*Bs* = 0.003–0.02, SE = 0.001–0.01, *ps* = 0.000–0.015).

Specifically, Figure 3B shows that the income return differences between females and males were small before expansion and increased after expansion, with males having a larger income penalty than females. Figure 3C showed that before expansion, urban residents had more income returns from overeducation; after expansion, in some cohorts, urban residents had the larger income penalty due to overeducation than rural residents. Figure 3D shows that before expansion, in many cohorts, people living in eastern areas had the highest income return from overeducation; however, after expansion, people living in middle areas slowly had the largest income penalty, while people living in western areas had the lowest income penalty from overeducation. Nonetheless, a reversal was observed in the two most recent cohorts, resulting in the lowest income penalty for residents in the eastern areas.

In Figure 3E, before the higher education expansion, participants whose parents were farmers or workers had the lowest income return, whereas after the higher education expansion, they had the lowest income penalty. In Figure 3F, within specific occupations, although income returns from overeducation fluctuate across cohorts, the overall trend remains relatively indistinct. However, after the expansion, clerks consistently exhibited the highest income returns from overeducation in many cohorts—the growth of manufacturing likely provided them with more opportunities, while elementary occupations, such as farm-hands and laborers, had the largest income penalty, suggesting a certain level of social inequality and a trapping effect. Moreover, participants who never worked full-time had the largest income penalty; nevertheless, after 1996–1999 cohorts, their income return became positive, which may be related to the recent rise of Internet platforms, which have enabled some young people to earn income by engaging in new types of occupations, such as live streaming.

In conclusion, after expansion, females, rural residents, people living in western areas, and participants whose parents were farmers or workers had lower income penalties than their counterparts, supporting H4b that disadvantaged groups (such as female or rural residents) have higher income returns than privileged groups (such as male or urban residents) after higher education expansion.

### 4.5 Robustness test

Mode method was used to calculate the year of overeducation as a robustness test. Results showed that cohort variances and its interaction variances were significant in all the models, suggesting significant cohort evidence on overeducation and income returns (see Supplementary material). Results were robust.

## 5 Discussion

### 5.1 Main findings

By comparing overeducation, its effect on income returns, and group disparities across 19 cohorts that entered the labor market before and after higher education expansion in China, using the Chinese General Social Survey from 2010 to 2021, this study traces the evolution of overeducation patterns, income, and social inequality in the context of higher education expansion.

First, the findings reveal significant cohort evidence of overeducation and income returns, consistent with other cohort studies (Artz and Welsch, 2021; Baran, 2025; Bar-Haim et al., 2019; Xu and Xu, 2025). Thus, the impact of higher education expansion and overeducation is persistent (Broniatowska, 2021). However, despite the expansion of higher education, other major historical events and policies may also have contributed to cohort differences. Prior to the expansion in China, the first peak of overeducation appeared around 1960–1965 cohorts, right at the intersection of the “Sent-Down Youth” movement and the restoration of the college entrance exams. During these cohorts, rural residents had a higher level of overeducation than urban residents, whereas after the restoration, urban residents started to have a higher level of overeducation. After the expansion, for younger cohorts born after 1998, they started to demonstrate distinct overeducation and income return patterns; their level of overeducation reduced, and rural residents had more years of overeducation than urban residents. Moreover, the income return of younger cohorts who never worked full-time increased positively. These may be driven by recent socioeconomic trends, such as the back-to-homeland wave, in which some university graduates choose to return to and contribute to their home villages (Xinhua, 2024), and the growth of the platform economy (e.g., live streaming).

Second, this study found that expansion led to a shift from a trade-off (income returns) to a trap (income penalty) associated with overeducation. This finding resonates with and extends the dynamic perspective of incomplete markets theory, which posits that labor markets undergo stages of adjustment following a supply shock, eventually reaching a point where excess qualifications are penalized. The inconsistent findings in the literature on overeducation and income returns may be attributed to variations in policy conditions and socioeconomic contexts across countries and periods. For instance, countries exhibit divergent patterns in overeducation and income returns (Chiroleu and Marquina, 2017; Di Stasio, 2017). In the context of our study, the observed income penalty may be explained by the fact that the expansion elevated the overall education level yet simultaneously intensified competition among individuals with similar qualifications and reduced the marginal benefit of higher education for employers. Consequently, companies may become less willing to offer premium wages to academically overqualified candidates, leading to penalties. Building on this finding, we further hypothesize that, in countries with larger-scale expansion, higher overall educational attainment, and more developed economies (since educational expansion may contribute to economic growth), the likelihood of experiencing an income penalty due to overeducation may be greater than that in other countries. This proposition, however, warrants explicit cross-national comparative investigation.

Therefore, these two main findings underscore the necessity of situating higher education expansion in a broader historical and political context. The effects of the expansion are not isolated; rather, they interact with other societal changes. In particular, as the expansion policy has been implemented for an extended period, an important avenue for future research is to examine whether and how its impact is attenuated or reshaped by concurrent structural changes.

Third, the study examined sex, rural–urban, regional, family background, and occupational disparities in overeducation and income. The results for over-education varied. The rural–urban and individual occupational differences showed that groups such as urban residents or higher occupational-status groups had a higher level of overeducation after expansion; while differences related to sex, region, and family background indicate that after expansion, groups such as female, people living in western China, and people whose parents were farmers/workers had to pursue more years of education to enter the same industry. However, regarding income return, the results were consistent in that after expansion, females, rural residents, people living in western areas, and participants whose parents were farmers or workers had lower income penalties than their counterparts, supporting the hypothesis that disadvantaged groups have higher income returns than privileged groups. These findings suggest that expansion has a sophisticated impact on income inequality through overeducation and differs according to group. A one-size-fits-all research theory may be inadequate; instead, more nuanced studies are needed to distinguish which specific group disparities widen or narrow. Furthermore, a one-size-fits-all policy is not suitable; it is necessary to develop targeted policies for distinct groups.

Fourth, overall, after the expansion, although females and people living in western areas have higher levels of overeducation, they have lower income penalties, suggesting a trade-off effect; rural residents have lower levels of overeducation and lower income penalties, also suggesting a trade-off effect. Therefore, in China, at group level, the expansion of higher education may have provided a compensatory mechanism, enabling disadvantaged groups to attain higher income levels by accessing more education, fulfilling their intended goal of reducing social inequality. Previous studies (e.g., Guo and Chen, 2023; Zhao, 2023), which predominantly focused on educational disparities exacerbated by expansion, offer an incomplete picture. Our findings suggest that educational inequalities may paradoxically translate into greater income equality. However, as enrollment continues to grow, educational attainment continues to rise, artificial intelligence increasingly replaces human labor, and fertility rates decline, it remains uncertain whether competition in the labor market will intensify or ease in the future. Thus, whether expansion will continue to mitigate or eventually exacerbate social inequality in China remains a critical question for future research.

### 5.2 Implications

This study yields several significant implications for policy formulation and future scholarly inquiry. First, the analysis suggests that the potential positive externalities of higher education expansion may outweigh its documented negative consequences in the Chinese context. Notably, the mechanism of overeducation itself may, under certain conditions, function as an inadvertent channel for reducing some forms of inter-group inequality—for instance, when it acts as a trade-off that provides disadvantaged groups a foothold in competitive labor markets. This finding challenges the negative perception of overeducation and aligns with scholars like Capsada-Munsech (2017), who caution that the term itself can be reductive, especially in an era championing lifelong learning. However, this does not negate the urgent need to address persistent and structural inequalities that the expansion process may have reconfigured rather than resolved. For example, the data indicate that women and rural residents often require higher educational credentials to secure positions comparable to those held by urban males, revealing a labor market that continues to impose discriminations on disadvantaged groups. Therefore, future research must move beyond simply measuring the incidence of overeducation to rigorously dissect its qualitatively different meanings and consequences across social strata.

Second, policymakers and researchers must adopt a systems-level perspective that acknowledges the interaction between educational expansion and other concurrent social and economic policies. The impact of expanding access is not determined in a vacuum but is mediated by the broader institutional environment, including industrial policy, regional development strategies, and social welfare systems. Comprehensive social governance is therefore essential to create an ecosystem where increased educational attainment translates into genuine human capital development and equitable opportunity. Future studies should explicitly model these policy interactions to identify synergistic or countervailing effects that shape the ultimate societal return on educational investment.

Third, policy design and evaluation must be fundamentally disaggregated and group-sensitive. Our findings clearly demonstrate that a uniform national policy, such as the 1999 expansion, generates heterogeneous effects. It may narrow certain opportunity gaps while inadvertently widening others, such as the quality-tier stratification within higher education or the urban–rural divide in high-status employment. Moving forward, policy planning should be informed by granular, group-specific analyses that can predict differential impacts. This necessitates a research agenda dedicated to identifying which specific group disparities are exacerbated or mitigated by expansion, enabling the design of targeted, compensatory interventions—such as affirmative action in elite university admissions, tailored career services for rural graduates, or lifelong learning subsidies for workers in declining industries—to ensure that the benefits of expansion are shared more equitably across society.

### 5.3 Contributions and limitations

This study contributes new evidence to the extensive discourse on the consequences of higher education expansion, particularly regarding overeducation and income returns. Theoretically, it advances the literature by operationalizing and empirically testing the core ‘trap versus trade-off’ dichotomy. This framework moves beyond debating whether overeducation is universally good or bad, and instead provides a dynamic lens to examine how its nature and consequence can transform under specific structural conditions, such as a large-scale supply shock.

By employing a rigorous cohort design to analyze the Chinese case, our analysis clarifies several key dynamics that help to contextualize and refine ongoing debates. First, it demonstrates that the impact of the expansion—and the overeducation it engendered—exhibits persistent cohort-level effects, suggesting that the policy’s influence extends beyond temporary market adjustments. Second, it highlights how the expansion’s outcomes are moderated by concurrent historical and policy contexts, implying that its effects cannot be understood in isolation. Third, and most centrally, it provides robust empirical support for a structural shift in the economic return to overeducation—from a potential trade-off associated with income gains in pre-expansion cohorts to a clear trap manifesting as an income penalty in post-expansion cohorts. Finally, it reveals that the effect of expansion and overeducation on income is heterogeneous across social groups; notably, in contemporary China, evidence suggests it may still operate partially as a trade-off for certain disadvantaged groups, even as the aggregate trend turns punitive.

Given these nuanced findings, our study offers valuable insights for other developing and East Asian economies undergoing similar educational transitions, while simultaneously underscoring the importance of national context in shaping outcomes. The generalizability of our conclusions is necessarily limited to settings comparable to China’s state-led, rapid expansion model. Future research should therefore prioritize cross-national comparative designs that can systematically test how varying institutional, economic, and policy environments condition the relationship between expansion, overeducation, and labor market inequality. Moreover, future research would benefit from incorporating pre-2010 and post-2021 data to examine how subsequent macro-shocks—including the full socioeconomic aftermath of the COVID-19 pandemic and other policy shifts—may interact with or alter the cohort trajectories identified here.

### 5.4 Conclusion

By comparing overeducation, its effect on income returns, and group disparities across 19 cohorts that entered the labor market before and after the higher education expansion in China using the Chinese General Social Survey from 2010 to 2021, the study had four main findings. First, the effect of higher education expansion and overeducation is persistent. Second, higher education expansion interacts with other historical events or policies. Third, expansion led to a shift from a trade-off (income returns) to a trap (income penalty) associated with overeducation. Finally, the impact of expansion and overeducation on income returns varies across different groups; nonetheless, in contemporary China, it predominantly functions as a trade-off effect.

## Data Availability

The datasets presented in this study can be found in online repositories. The names of the repository/repositories and accession number(s) can be found at: http://cgss.ruc.edu.cn/English/Home.htm.

## Footnotes

1 For more information about CGSS, please visit the official CGSS website at http://cgss.ruc.edu.cn/English/Home.htm.
