# Supplementary material for: Trap versus trade-off: cohort evidence on overeducation, income returns, and group disparities after China’s higher education expansion
Source: Front Sociol. 2026 Feb 18;11:1705158. doi: 10.3389/fsoc.2026.1705158 (PMC12956667; doi:10.3389/fsoc.2026.1705158)
Supplement: Supplementary file 1 [file Table_1.docx]

**Supplementary Table 1. Random effects of overeducation.**

| variance | model 1 | model 2 | model 3 | model 4 | model 5 | model 6 | model 7 | model 8 |
| --- | --- | --- | --- | --- | --- | --- | --- | --- |
| period | 0.03 (0.02) | 0.02 (0.01) | 0.01 (0.01) | 0.01 (0.01) | 0.01 (0.01) | 0.01 (0.01) | 0.01 (0.01) | 0.06 (0.04) |
| cohort | 0.34^**^ (0.14) | 0.33^**^ (0.13) | 0.35^**^ (0.14) | 0.35^**^ (0.15) | 0.30^**^ (0.12) | 0.34^**^ (0.14) | 0.29^**^ (0.13) | 0.0004 (0.02) |
| gender × cohort |  |  |  | 0.15^*^ (0.07) |  |  |  |  |
| rural-urban × cohort |  |  |  |  | 0.08^*^ (0.04) |  |  |  |
| living area × cohort |  |  |  |  |  | 0.05^**^ (0.02) |  |  |
| parents' occupation × cohort |  |  |  |  |  |  | 0.03^**^ (0.01) |  |
| individual occupation × cohort |  |  |  |  |  |  |  | 0.40^***^ (0.06) |
| residual | 12.81^***^ (0.08) | 11.81^***^ (0.07) | 11.80^***^ (0.07) | 11.78^***^ (0.07) | 11.79^***^ (0.07) | 11.78^***^ (0.07) | 11.79^***^ (0.07) | 11.80^***^ (0.12) |
| BIC | 274528.5 | 270446.7 | 270445.9 | 270391.1 | 270398.5 | 270378.9 | 270422.1 | 251991.4 |

Note. Overeducation was calculated by mode method. Model 1 was the model without control variables; model 2 was controlled for individual variables; model 3 ~ 8 were controlled for individual and macro socioeconomic variables. ^*^ *p* < 0.05; ^**^ *p* < 0.01; ^***^ *p* < 0.001.

**Supplementary Table 2. Random effects of overeducation on income returns.**

| variance | model 1 | model 2 | model 3 | model 4 | model 5 | model 6 |
| --- | --- | --- | --- | --- | --- | --- |

| period | 0.11 (0.10) | 0.11 (0.11) | 0.11 (0.11) | 0.11 (0.10) | 0.11 (0.11) | 0.25^*^ (0.14) |
| --- | --- | --- | --- | --- | --- | --- |
| cohort | 0.75^*^ (0.36) | 0.76^*^ (0.37) | 0.74^*^ (0.36) | 0.76^*^ (0.37) | 0.74^*^ (0.36) | 0.01 (0.01) |
| overeducation × cohort | 0.01^**^ (0.004) | 0.01^**^ (0.005) | 0.01^**^ (0.01) | 0.01^**^ (0.004) | 0.01^*^ (0.004) | 0.0009 (0.001) |
| gender × overeducation × cohort |  | 0.01^*^ (0.004) |  |  |  |  |
| rural-urban × overeducation × cohort |  |  | 0.01^**^ (0.003) |  |  |  |
| living area × overeducation × cohort |  |  |  | 0.001^*^ (0.0007) |  |  |
| parents' occupation × overeducation × cohort |  |  |  |  | 0.003^**^ (0.001) |  |
| individual occupation × overeducation × cohort |  |  |  |  |  | 0.006^***^ (0.002) |
| Residual | 14.32^***^ (0.09) | 14.29^***^ (0.09) | 14.28^***^ (0.09) | 14.31^***^ (0.09) | 14.30^***^ (0.09) | 12.46^***^ (0.08) |
| BIC | 280368.1 | 280273.8 | 280272.5 | 280356.4 | 280325.6 | 269522.6 |

Note. Overeducation was calculated by mode method. Individual and macro socioeconomic variables were controlled. ^*^ *p* < 0.05; ^**^ *p* < 0.01; ^***^ *p* < 0.001.
